# Development and validation of a biological risk assessment tool among hospital personnel under COVID-19 pandemic conditions

**DOI:** 10.1371/journal.pone.0286298

**Published:** 2023-05-30

**Authors:** Amir Hossein Khoshakhlagh, Saeid Yazdanirad, Hamid Reza Saberi, Masoud Motalebi Kashani, Fatemeh Ghanaei Khaledabadi, Fazel Mohammadi-Moghadam, Muhammet Gul

**Affiliations:** 1 Department of Occupational Health, School of Health, Kashan University of Medical Sciences, Kashan, Iran; 2 Social Determinants of Health Research Center, Shahrekord University of Medical Sciences, Shahrekord, Iran; 3 School of Health, Shahrekord University of Medical Sciences, Shahrekord, Iran; 4 Occupational Health & Safety Department, Kashan University of Medical Sciences, Kashan, Iran; 5 Social Determinants of Health Research Center, Kashan University of Medical Sciences, Kashan, Iran; 6 Department of Environmental Health Engineering, School of Health, Shahrekord University of Medical Sciences, Shahrekord, Iran; 7 Dept. of Transportation and Logistics, Istanbul University, Avcılar/Istanbul, Turkey; The Maldives National University, NEPAL

## Abstract

The need for a biological disease risk assessment method to prevent the contagion of these diseases, particularly among healthcare personnel, is crucial. Therefore, this study aimed to develop and validate a biological risk assessment tool for biological agents among hospital personnel under COVID-19 conditions. This cross-sectional study was performed on 301 employees in two hospitals. Firstly, we identified the items affecting the contagion of biological agents. Then, we computed the weight of the items using the Fuzzy Analytical Hierarchy Process (FAHP) method. We used the identified items and the estimated weights in the next step to develop a predictive equation. The outcome of this tool was the risk score of biological disease contagion. After that, we used the developed method to evaluate the biological risk of the participants. The ROC curve was also used to reveal accuracy of developed method. In this study, 29 items were identified and categorized into five dimensions, including environmental items, ventilation items, job items, equipment-related items, and organizational items. The weights of these dimensions were estimated at 0.172, 0.196, 0.255, 0.233, and 0.144, respectively. The final weight of items was used to develop a predictive equation. The area under ROC curves (AUC) was also calculated as 0.762 (95% CI: 0.704, 0.820) (p<0.001). The tools developed using these items had acceptable diagnostic accuracy for predicting the risk of biological diseases in health care. Therefore, one can apply it in identifying persons exposed to dangerous conditions.

## Introduction

Occupational hazards are major occupational health problems that can cause workplace accidents or illnesses [1]. Hospital is one of the high-risk environments in which working people are exposed to occupational hazards [2]. One of the significant occupational hazards among healthcare personnel is biological hazards. Exposure to biological agents in the workplace increases the risk of infectious diseases [3]. These infections are caused by contact with microbes in various ways [3, 4].

There are risk factors affecting the contagion of biological diseases in hospitals. Some critical factors are the presence of infected people inwards, management status of waste such as contaminated clothing or equipment, and cleaning status of surfaces and facilities. A sick person or contaminated clothing, equipment, and surfaces can be the source of the spread of microorganisms in the hospital environment [5]. Another important group of factors is the status of ventilation in the environment. Natural and artificial ventilation, heating-cooling systems, and air filtration system in hospitals also affect the spread of many pathogens [5]. Factors such as the crowdedness of hospital wards and their poor service quality, the large number of patients admitted to wards, long staying of visitors in the wards, and high humidity of the air leading to the growth of microorganisms on the surfaces are other factors affecting air pollution [6]. It is recommended for healthcare personnel in the hospital to use personal protective equipment while working [7]. Therefore, the use and efficiency level of personal protective equipment can also impress on the contagion of biological diseases [7]. Also, compliance with health protocols and organizational surveillance can control the occurrence of this disease. Hence, those can be considered effective agents [8].

Coronavirus has been one of the critical biological agents in the last two years [9]. It appeared on December 31, 2019, in Wuhan, China [10]. This disease have killed more than 5 million people worldwide [11]. Therefore, the prevention of this disease is vital. For this purpose, it will be useful to predict the probability of the disease by a risk assessment tool before its occurrence in the individuals using the items affecting the disease contagion and to focus on the control of important items for preventing the disease [12].

A risk assessment tool is one of the basic steps in all occupational safety and health management systems to identify, assess, and prioritize occupational hazards [13]. Previous studies have also tried developing a predictive biological risk assessment model. Ghayvat et al. developed a tool for predicting the spread of contagion based on mobile phone location data. This system could identify the COVID-19 infectious and hazardous sites and detect disease outbreaks [14]. Sangiorgio and Parisi also developed a useful tool using artificial neural networks to predict the risk of contagion using socio-economic data, such as the presence of activities, companies, institutions, and the number of infections in urban districts [15]. Buonanno et al. studied the quantitative assessment of the risk of airborne transmission of SARS-CoV-2 disease [16]. Cabarkapa et al. also investigated psychosocial stress and risk assessment during the COVID-19 pandemic [17]. However, a limited number of risk factors have been investigated in that study. Also, some previous tools used complex analysis, such as artificial neural networks, which restrict their applicability.

There is no simple and comprehensive tool to evaluate the probability of contagion of biological agents using various risk factors among healthcare personnel. Therefore, this study aimed to develop and validate a biological risk assessment tool for biological agents among hospital personnel under COVID-19 pandemic conditions.

## Material and method

This cross-sectional study was carried out in two phases, development and validation of the tool, in 2022. The data was collected using the developed tool. The protocol was reviewed and approved by the Medical Ethics Committee of Kashan University of Medical Sciences (IR.KAUMS.NUHEPM.REC.1400.055). All steps of the study were in accordance with the ethical standards. All participants were asked to fill out the consent form developed by the medical ethics committee, and written informed consent was obtained from all of them.

### Participants

In the tool development step, 15 experts performed pairwise comparisons. Five nurses, seven physicians, and three occupational health professors participated in this step. The inclusion criteria for these individuals included having a history of research on the contagion of biological agents and knowing the factors affecting this contagion. On the other hand, the exclusion criterion was an unwillingness to participate in the study. These persons were selected by convenience Sampling based on the inclusion criteria. In the validation step, we assessed the contagion risk of COVID-19 disease for 301 employees of two Iranian hospitals. The inclusion criteria for these subjects consisted of having work experience higher than one year in a hospital. The exclusion criterion also included unwillingness to participate in the study. For selection of the samples, a list of the nurses occupied in COVID-19 ward in two these hospitals, including 523 nurses and physicians, was prepared. Then, 400 individuals were randomly selected from them. After that, their medical records were reviewed. Following that, 368 nurses with inclusion criteria were entered the study. Among them, 301 nurses accepted to participate in the study. The situation of 228 nurses and 73 physicians was examined in this step. Of them, 180 persons had a history of COVID-19 in the last year, and 121 had no record of this disease.

### Sample size

Given the aim of this study, a minimum value of the correlation coefficient between the predictive score of disease risk and the actual status of the disease was considered 0.2. The sample size with a confidence level of 95% and a test power of 90% was computed as in Eq (1):

$$n=\frac{{\left(Z_{1-\frac{\alpha }{2}}+Z_{1-\beta }\right)}^{2}}{w^{2}}+3≅259$$ (1)

Where $Z_{1-\frac{\alpha }{2}}$ is equal to 1.96 based on a confidence level of 95%, Z_1-β_ is equal to 1.29 based on a test power of 90%, and *w* is equal to 0.203 based on a minimum correlation coefficient of 0.2. Therefore, we suggested the sample size in the present study be more than 259.

### The development of the tool

#### Identifying items affecting the contagion of biological agents

At this stage, a non-systematic review was performed using various keywords in valid databases such as ISI, PubMed, Scopus, Science Direct, and Google Scholar. The factors affecting the contagion of biological agents were identified. Two groups of keywords were used in this study. The first group includes the keywords “factor, risk factor, agent, item, relationship, prediction, association, and associated”. Key words of the second group consist of “contagion disease, biological, contagious disease, coronavirus, and COVID”. Unrelated and duplicated items also were removed. Then, the identified literature was reviewed, and the items were extracted. The total items, such as ventilation, job, personal protective equipment, organizational items, and environmental items, were used as the keywords for a second search. These items were considered predictors for estimating the risk of the contagion of biological agents.

#### Weighting the effective items

The effective items’ relative and final weight were estimated using the Fuzzy Analytical Hierarchy Process (FAHP) method. For this purpose, a hierarchical structure was first depicted. In the next step, tables of pairwise comparisons between items and dimensions were provided and sent to 20 experts through electronically, of which 15 persons filled out the forms. They compared the items in terms of relative importance in the contagion of biological agents using a five-point Likert scale (1, 3, 5, 7, and 9). This scale is based on linguistic expressions (same importance, slightly more important, more important, very important and extremely more important). The weight of the agents was computed using Chang’s extent analysis method on FAHP. In this method, linguistic words are transformed into triangular fuzzy numbers and the weight of the items is estimated by the relevant equations so that uncertainties in pairwise comparisons are decreased [18]. Triangular fuzzy numbers were applied to weigh the experts^’^ opinions. Therefore, the linguistic words selected by experts are transformed into triangular fuzzy numbers. These are three numbers between 0 and 1, shown as *M* = (*l,m,u*). In this equation, *l*, *m*, and *u* are the lower, medium, and upper values of the triangular fuzzy number. After that, the relative weight of each item is calculated by these fuzzy numbers based on the Chang et al. method [18].

#### Developing novel tool and predictive equation

Final weights related to the effective items, calculated in the previous step, were used to develop the risk equation. In this way, these weights were considered as the coefficients of items. In other words, to calculate the total score in a novel equation, each of these coefficients was multiplied by the score of the related item, and the resultant values were summed together. A five-point Likert scale from 0 to 4 is assigned to the score of each item based on its situation. The final score computed by the predictive equation was considered the outcome of this study. A higher final score shows a higher probability of disease contagion.

### The validation of the tool

#### Determining risk levels and the method’s validation

SARS-CoV-2 was considered a biological agent. In this study, we evaluated the risk of SARS-CoV-2 contagion. For this purpose, the risk of disease for 301 healthcare personnel in different wards of two hospitals in Kashan in 2022 was estimated using the designed tool. Also, the contagion history of these people to COVID-19 in the last year (identified by PCR test as the gold standard) was gathered. There is a cycle between status of items affecting the contagion and real contagion. The conditions evaluated by these items cause the contagion of COVID-19. These nearly constant conditions have caused disease contagion in the medical staff during the last year. The ROC curve was used to reveal the appropriate cut-off point and validate the developed tool as a method predictive of risk related to COVID-19 diseases.

### Statistical analysis

The weights of the items as predictors were computed in the MS Excel following the FAHP algorithm to develop the tool. To validate the tool, the collected data was entered into SPSS software. The expectation maximization method was applied to calculate and replace the missing values. Then, the descriptive analysis was performed. Moreover, ROC analysis was performed to validate the developed tool and determine the cut-off point of the risk levels.

## Results

### Participants

Table 1 represents the demographic characteristics of the participants.

**Table 1:** Demographic characteristics of the participants.

Step	Variable	Range	Mean	Standard variation
Development of the tool	Age (year)	19.00–54.00	31.73	6.84
	Work experience (year)	1–25	7.12	7.54
	Height (meter)	1.52–1.95	1.68	8.39
	Weight (kilogram)	62.00–94.00	70.24	33.93
Validation of the tool	Age (year)	30.00–61.00	43.27	10.52
	Work experience (year)	5–31	13.28	9.76
	Height (meter)	1.59–1.89	1.72	6.39
	Weight (kilogram)	57.00–86.00	68.14	15.91

### The development of the tool

#### Items affecting the contagion of biological agents

In this study, 29 items were identified and categorized into five groups, including environmental items, ventilation items, job items, equipment-related items, and organizational items. Environmental items include the cleaning status of surfaces, separation status of hazardous wards, crowd in a ward, presence of visitors, availability status of health facilities, cleaning status of health facilities, and status of waste management. Ventilation items consisted of the quality of natural air conditioning, the quality of artificial air conditioning, the filtration status of the ventilation system, and the status of ultraviolet light use in the ventilation system. Job items included the close contact with patients, the danger level of a person’s job, the presence in dangerous wards, the status of compliance with health protocols by personnel, and the status of compliance with health protocols by visitors. Equipment-related items consisted of the availability status of personal protective equipment, usage of personal protective equipment, the replacement of contaminated equipment or clothing, suitability of personal protective equipment with the desired job, and efficiency level of used personal protective equipment. Organizational items comprise education level of health principles, surveillance level on compliance with health protocols, surveillance level on identification and quarantine of infected cases, the status of encouragement—punishment system for compliance with health protocols, prolonged work shifts, and vaccination of personnel.

#### Weight of effective items

FAHP was applied to the weight of each of the effective items and dimensions in the contagion of biological agents. This study’s consistency ratio (CR) was 0.075, which is acceptable. Table 2 reports the relative weight and prioritization of the dimensions. Based on the results, the dimension of job items (0.255) and equipment-related items (0.233) had the highest relative weight and ranking order, respectively. Other preferences were assigned to ventilation, environmental, and organizational items. Table 3 shows the relative weight of effective items in each dimension. Based on the results, the separation status of hazardous wards (0.198) and the crowd in the ward (0.172) have the highest relative weight among all environmental items, respectively. Of the ventilation items, the status of artificial (0.273) and natural (0.268) air conditioning have the highest relative weights, respectively. Of the job items, danger level of person’s job (0.221) and presence in dangerous wards (0.213) are determined as the first and second priorities, respectively. Among all equipment-related items, the efficiency level of used personal protective equipment (0.211) and suitability of personal protective equipment with the desired job (0.208) have the first and second ranking order, respectively. Of organizational items, surveillance level on identification and quarantine of infected cases (0.184), and status of encouragement—punishment system for compliance with health protocols (0.175) are placed in the first two rank, respectively.

**Table 2:** The relative weight and ranking order of the dimensions.

Parameters	Relative weight	Rank
Environmental items	0.172	4
Ventilation items	0.196	3
Job items	0.255	1
Equipment-related items	0.233	2
Organizational items	0.144	5

**Table 3:** The relative weight and prioritization of effective items in each dimension.

Parameters			Relative weight	Final weight	Rank
Environmental items	V1	Cleaning status of surfaces	0.145	0.025	4
	V2	Separation status of hazardous wards	0.198	0.034	1
	V3	Crowd in ward	0.172	0.030	2
	V4	Presence of visitors	0.168	0.029	3
	V5	Availability status of health facilities	0.103	0.018	6
	V6	Cleaning status of health facilities	0.101	0.017	7
	V7	Status of waste management	0.113	0.019	5
Ventilation items	V8	Air temperature	0.158	0.031	5
	V9	Air humidity	0.152	0.030	6
	V10	Status of natural air conditioning	0.182	0.036	2
	V11	Status of artificial air conditioning	0.186	0.036	1
	V12	Filtration status of the ventilation system	0.162	0.032	3
	V13	Status of ultraviolet light use in the ventilation system	0.160	0.031	4
Job items	V14	Close contact with patients	0.202	0.051	3
	V15	Danger level of a person’s job	0.221	0.056	1
	V16	Presence in dangerous wards	0.213	0.054	2
	V17	Status of compliance with health protocols by personnel	0.184	0.047	4
	V18	Status of compliance with health protocols by visitors	0.180	0.046	5
Equipment-related items	V19	Availability status of personal protective equipment	0.193	0.045	4
	V20	Use of personal protective equipment	0.204	0.048	3
	V21	Replacement of contaminated equipment or clothing	0.184	0.043	5
	V22	Suitability of personal protective equipment with the desired job	0.208	0.048	2
	V23	Efficiency level of used personal protective equipment	0.211	0.049	1
Organizational items	V24	Education level of health principles	0.146	0.021	6
	V25	Surveillance level on compliance with health protocols	0.172	0.025	3
	V26	Surveillance level on identification and quarantine of infected cases	0.184	0.026	1
	V27	Status of encouragement—punishment system for compliance with health protocols	0.175	0.025	2
	V28	Prolonged work shifts	0.157	0.023	5
	V29	Vaccination of personnel	0.166	0.024	4

#### Developed tool and predictive equation

S1 Table in S1 File represents the guidance for scoring the items in the developed tool. Indeed, using this tool, an expert evaluates each item based on a five-point Likert scale.

The final weight of items, computed in the previous stage, was used to develop a predictive equation. Each of these weights is multiplied by the score of the related item, and the resultant values are summed together to calculate the total score of the novel equation.

BRA = [(0.024*v1)+(0.034*v2)+(0.030*v3)+(0.029*v4)+(0.018*v5)+(0.017*v6)+(0.019*v7)+(0.031*v8)+(0.030*v9)+(0.036*v10)+(0.036*v11)+(0.032*v12)+(0.031*v13)+(0.051*v14)+(0.056*v15)+(0.054*v16)+(0.047*v17)+(0.046*v18)+(0.045*v19)+(0.048*v20)+(0.043*v21)+(0.048*v22)+(0.049*v23)+(0.021*v24)+(0.025*v25)+(0.026*v26)+(0.025*v27)+(0.023*v28)+(0.024*v29)] × 100 (2)

Where v1 to v29 are scores of the items evaluated in the novel tool.

### The validation of the tool

#### Risk levels and method validation

**Fig 1:**
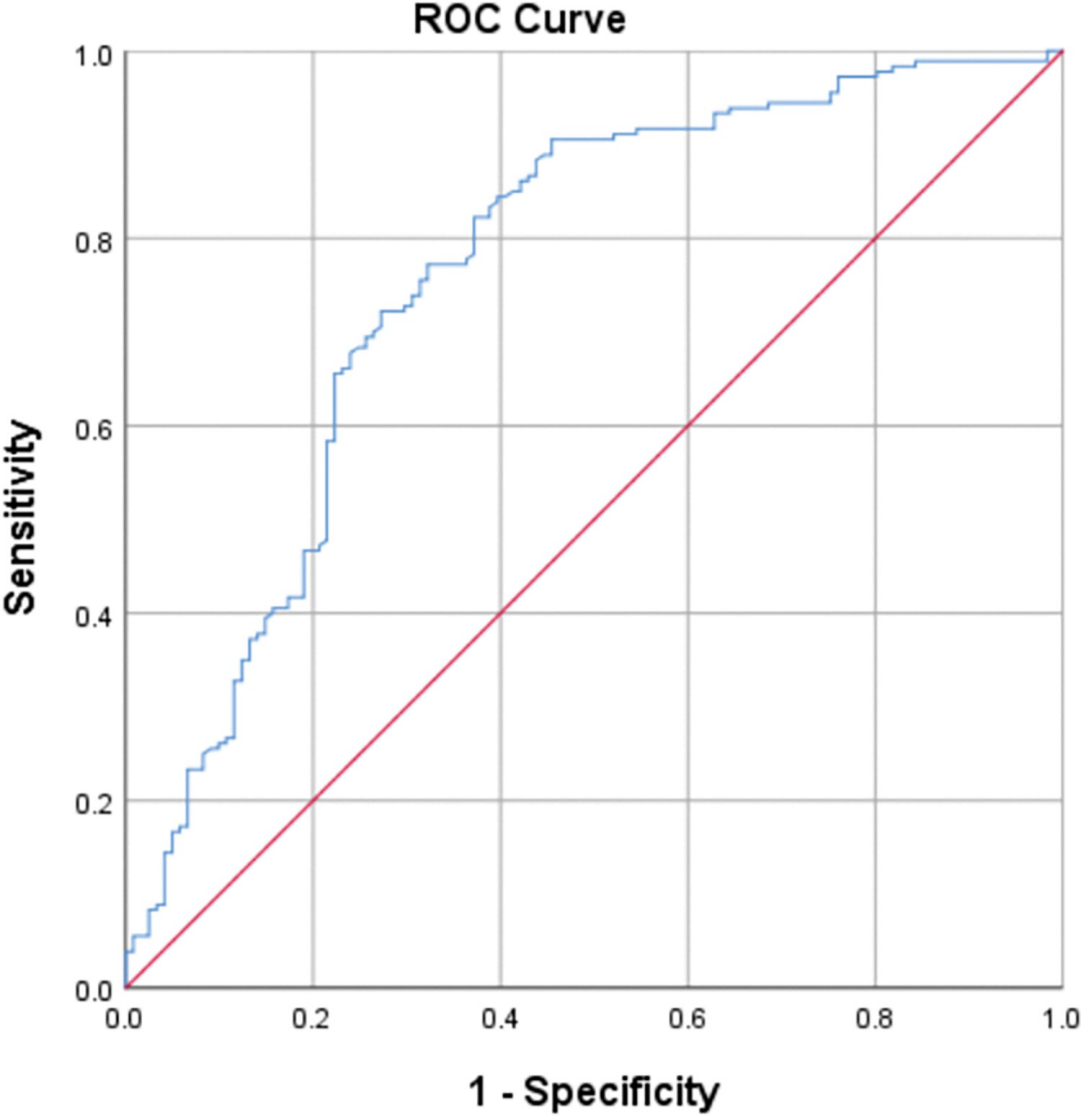
Receiver operating characteristic (ROC) curve.

The method developed based on the items affecting the contagion of biological agents was applied to evaluate the biological risk of employees in two hospitals. The ROC curve was used to reveal the appropriate cut-off point and validate the developed method as a tool predictive of risk related to COVID-19 diseases. In Fig 1, where the ROC curve is exhibited, the point with the highest sensitivity and specificity has been considered the best cut-off point. The results indicate that the optimal cut-off point between low and high risk is equal to 255.45 (sensitivity = 0.772 and specificity = 0.678). It means that if the value resultant of the BRA equation is lower than 255.45, the risk is low; and if it is higher than 255.45, the risk is high. The area under ROC curves (AUC) was also calculated as 0.762 (95% CI: 0.704, 0.820) (p<0.001). The AUC was greater than 0.70 indicates acceptable diagnostic accuracy of the developed method.

## Discussion

In this study, 29 items affecting the contagion of biological agents were identified and categorized into five groups, including environmental items (7 parameters), ventilation items (6 parameters), job items (5 parameters), equipment-related items (5 parameters), and organizational items (6 parameters). In the previous literature, various studies have studied the effective items on the occurrence of biological diseases. Wang et al. examined the effective factors associated with the transmission of SARS-CoV-2 among healthcare workers in Wuhan, China. In that study, five critical factors were identified including touching the eyes, nose, and mouth while working, having appropriate personal protective equipment, having a history of presence in dangerous places, presence in crowded places, and contact with wildlife [19]. In a cross-sectional study in 2020 by Hussen et al., factors associated with SARS-CoV-2 infection among healthcare workers have been studied. In that research, six effective factors were recognized including the danger level of the job, prolonged working hours, an insufficient supply of personal protective equipment, lack of access to alcohol, non-observance of proper distance from patients, and close and direct contact with patients [11]. However, these studies have investigated a limited number of effective items. The results show that a higher number of effective items were identified in the current study compared to previously published studies. Indeed, in this study, the factors revealed in various studies were gathered. Then, they were comprehensively categorized into five groups based on similar properties.

The present study revealed that the dimension of job items had the highest relative weight and ranking order among all dimensions. The results of other studies also indicate the importance of the type of person’s job on the transmission of biological disease. The higher the danger level of an individual’s job in terms of exposure to infected patients and contaminated equipment are associated more probability of contagion of the biological diseases to them. If the danger level of a person’s job is low, importance level of other dimensions decreases. The highest weight is assigned to the job dimension in our study. Mutambudzi et al. concluded that occupation type affects the risk of severe COVID-19. They observed that essential workers have a higher risk of this disease. So medical support staff (RR = 8.70), social care (RR = 2.46), and transport workers (RR = 2.20) had the highest risk among occupational groups [20]. Moreover, results of Guo et al.’s study showed that the aerosol and surface distribution of severe acute respiratory syndrome SARS-CoV-2 II are different in various wards of a hospital in Wuhan, China. Based on the results of that study, contamination is greater in intensive care units compared to general wards [21]. Therefore, the employee of these wards exposes to a higher risk of Covid-19 disease. However, there are effective dimensions that their importance should be not ignored. Because the use of these dimensions can help to make risk assessment more accurately.

Based on the results of the present study, the separation status of hazardous wards and the crowd in ward got the highest relative weight among all environmental items, respectively. Separating the hazardous wards from general wards is a useful solution for preventing the transition of biological agents. The results of a study performed by Wan et al. revealed that the distribution of SARS-CoV-2 on the surfaces and air in various wards is different [22]. Therefore, if dangerous parts are not separate, the virus is freely transmitted to various places and more populations expose to it. The crowd in a ward was another important factor. The presence of many persons in indoor environments increases the probability of transition of the biological agents between humans. Burge et al. concluded that the crowd in ward is one of the important parameters affecting air pollution to microorganisms [6]. Of the ventilation items, the status of artificial and natural air conditioning had the highest relative weights, respectively. The results of Stockwell et al.’s study showed that advanced mechanical ventilation systems in hospitals help improve indoor air quality and reduce the risk of transmitting infectious diseases [23]. Moreover, Park et al. concluded that natural ventilation has a significant effect on airborne transmission in the building and it can be effective in preventing COVID-19 [24]. Of the job items, the danger level of person’s job and presence in dangerous wards were selected as the first and second priorities, respectively. It is clear that increasing danger level of job and presence in dangerous ward are associated with higher risk of disease because of the greater distribution of biological agents in the air and on the surfaces. Therefore, high weight of these two items seems logical. Because if the danger level of person’s job is low, the importance of other items decreases. In many studies, usage of personal protective equipment is considered useful in preventing biological disease, particularly in healthcare workers [25]. However, type of personal protective equipment used is important so that this equipment must be possessed enough efficiency against biological agents. Otherwise, the use of personal protective equipment will be futile [26]. Moreover, access to this equipment is important and if they are not available in the workplace, people are at biological risk. In total, results of several studies indicate that compliance with health protocols had a significant impact on controlling transmission of microorganisms [27]. In workplaces such as hospitals, organizational surveillance and rules can be helpful in performing these protocols.

A tool of biological risk assessment was designed using these items and the weights estimated for those were used to obtain a risk equation. The risk score resultant from this equation was categorized into low and high-risk levels. The results of ROC analysis showed that the developed tool has proper sensitivity and specificity in determining the risk of biological diseases. Also, this tool possesses an acceptable diagnostic accuracy. Therefore, it can be used to predict the probability of a biological disease, particularly, in healthcare personnel. One of the limitations of this study is regarding lack of examining validation of the developed method in evaluating the risk of other biological diseases.

## Conclusion

In total, 29 items affecting contagion of biological agents were identified and grouped into five dimensions: environmental items, ventilation items, job items, equipment-related items, and organizational items. The results showed the dimension of job items has the highest relative weight and rank among all dimensions. Based on the results, the tools developed using these items have an acceptable diagnostic accuracy for predicting the risk of biological diseases in healthcare. Therefore, it can be applied to identify persons exposed to risky conditions and help managers performing preventive measures. Moreover, this tool can be used for revealing important items in each workplace. For more validation of the developed tool, it was suggested a longitude study in the future researches in other working environments.

## Supporting information

S1 File The manuscript contains all the supporting tables and figures. (DOCX)

## Abbreviations

FAHP: Fuzzy Analytical Hierarchy Process
AUC: area under of ROC curves
BRA: biological risk assessment
ROC: receiver operator curves
