# Supplementary material for: Development and validation of a biological risk assessment tool among hospital personnel under COVID-19 pandemic conditions
Source: PLoS One. 2023 May 30;18(5):e0286298. doi: 10.1371/journal.pone.0286298 (PMC10228796; doi:10.1371/journal.pone.0286298)
Supplement: S1 File — (DOCX) [file pone.0286298.s001.docx]

**Supporting Information**

The manuscript contains all the supporting tables and figures
